# Model of Success: World Association for the Advancement of Veterinary Parasitology African Foundation (1997–2019)

**DOI:** 10.4102/jsava.v91i0.2019

**Published:** 2020-08-31

**Authors:** Rosina C. Krecek, Annemarie Avenant-Oldewage, Maggie Fisher, Barend L. Penzhorn, Isaac K. Phiri, Roger Prichard, Derek Sumption, Stephen R. Werre

**Affiliations:** 1 Private, College Station, Texas, United States; 2 Department of Zoology, University of Johannesburg, Auckland Park, South Africa; 3 Shernacre Enterprise Ltd., Malvern Worcs, United Kingdom; 4 Department of Veterinary Tropical Diseases, University of Pretoria, Onderstepoort, South Africa; 5 School of Veterinary Medicine, University of Zambia, Lusaka, Zambia; 6 Institute of Parasitology, Quebec, Canada; 7 Brantam House, Craighall, South Africa; 8 Population Health Sciences, Virginia-Maryland Regional College of Veterinary Medicine, Virginia, United States

## Introduction

**FIGURE 1:**
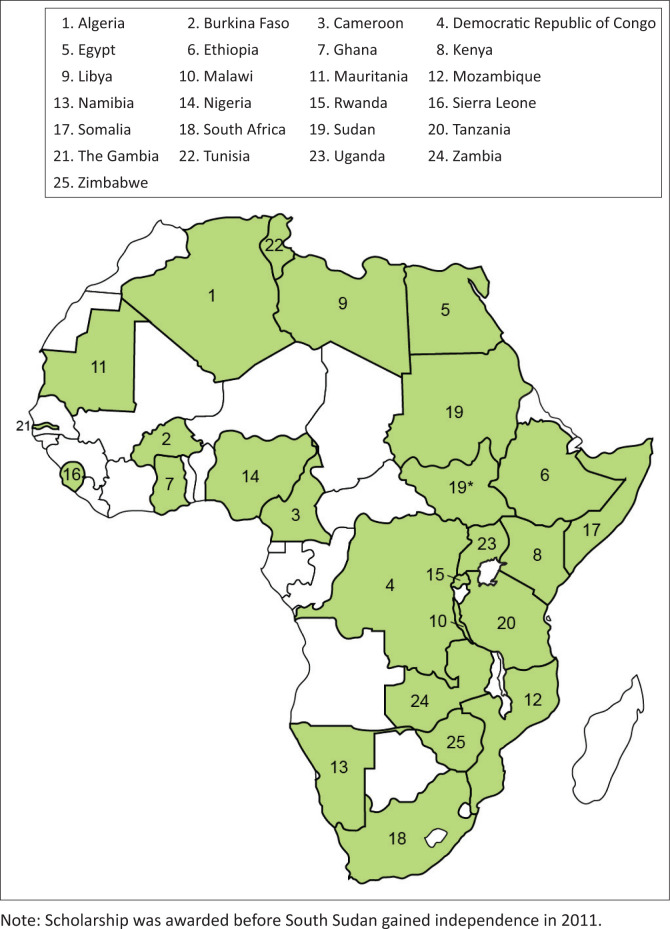
A total of 211 next-generation African veterinary parasitologists from 25 African countries submitted applications for the World Association for the Advancement of Veterinary Parasitology African Foundation Scholarships. Eighty scholarships were awarded to applicants from 18 countries (number of applicants): Algeria (2), Burkina Faso (1), Egypt (1), Ethiopia (2), Kenya (16), Mozambique (1), Nigeria (10), Sierra Leone (1), Somalia (1), South Africa (26), South Sudan (1), Sudan (3), Tanzania (4), The Gambia (1), Tunisia (2), Uganda (2), Zambia (5) and Zimbabwe (1).

**FIGURE 2:**
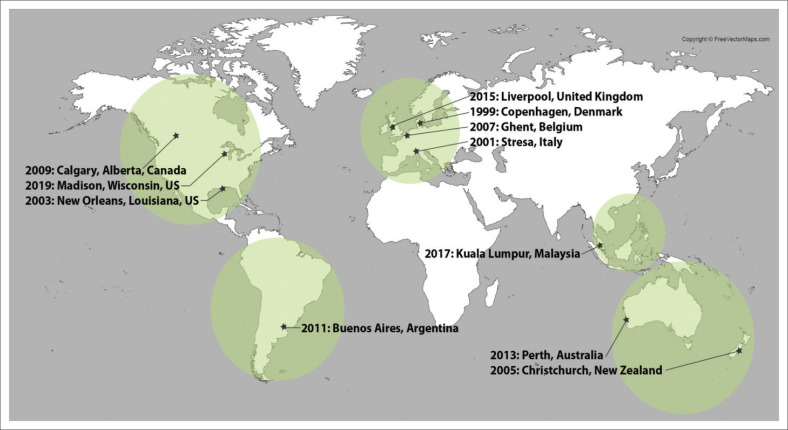
World Association for the Advancement of Veterinary Parasitology African Foundation awardees have presented their research at 11 International World Association for the Advancement of Veterinary Parasitology conferences globally from 1999 to 2019 (number of awardees). 2019: Madison, WI, United States (7); Kuala Lumpur, Malaysia (5); 2015: Liverpool, United Kingdom (7); 2013: Perth, Australia (8); 2011: Bueno Aires, Argentina (4); 2009: Calgary, Canada (10); 2007: Ghent, Belgium (8); 2005: Christchurch, New Zealand (4); 2003: New Orleans, LA, United States (10); 2001: Stresa, Italy (8); 1999: Copenhagen, Denmark (7).

In 1997, the World Association for the Advancement of Veterinary Parasitology African Foundation (WAAVP AF) achieved a ‘first’ by establishing a successful endowment which awards travel scholarships to next-generation (NG) African veterinary parasitologists to present their research findings at the WAAVP Biennial Conferences (e.g. ed. Colwell 2009; Krecek et al 2011; Krecek et al 2013; Krecek et al 2019; ed. Lymbery 2013; eds. Matthews, Williams & Trees 2015; eds. Prichard & Steffan 2011). To date, 211 NG veterinary parasitologists from 25 African countries submitted applications (Figure 1), and through a competitive selection, 80 applicants have been awarded from 18 countries to present their research at 11 WAAVP conferences in Europe, Asia, South America, the United States, Canada, Australia and New Zealand (Figure 2).

The WAAVP AF goals are threefold: to support deserving awardees ready to present their research to an international audience; to develop global networks; and to bring positive recognition to their institutions, countries and academic fields. A six-member WAAVP AF Committee oversees a successful financial investment and promotes these travel scholarships to more than 100 global institutions. The Committee members represent the WAAVP, Parasitological Society of Southern Africa and other research affiliations in Africa. The scholarship announcement is circulated to the WAAVP Secretariat and posted on the website (https://www.waavp.org). To promote this scholarship, WAAVP AF announcements have been translated into four languages of English, French, Arabic and Portuguese. The WAAVP African Foundation Biennial Reports and Financial Statements are submitted and presented to the WAAVP Executive Committee.

The importance of fostering NG veterinary parasitologists to be exposed to the impact of this field is crucial for the future. Travel scholarship recipients are encouraged to publish their research findings in high-impact peer-reviewed scientific journals in Africa as well as abroad. Using two on-line databases, the published research output of all awardees was documented. This publication highlights the WAAVP AF veterinary parasitologists and their commitment to identifying innovative and novel solutions to address parasitic challenges.

Worldwide there are numerous parasitology resources available, including organisations and societies. Over 100 are included on the websites of the American Society of Parasitologists (https://www.amsocparasit.org) and the British Society of Parasitology (http://bsp.uk.net/). In addition, the World Health Organization (WHO) (https://www.who.int) and Centers for Disease Control and Prevention (CDC) (https://www.cdc.gov) list many public health and funding agencies.

## Materials and methods

From 2007 to 2017, 42 awardees participated in WAAVP conferences, and 37 submitted narratives published in the WAAVP Quarterly Online Newsletters (for 21st–26th Conferences, https://www.waaavp.org). Data from 2007–2017 WAAVP African Foundation scholarship recipients who submitted online newsletters were analysed to include newslettter publication dates, conferences attended, recipient’s gender, and home country (Table 1). These narratives were extracted from the newsletters and converted into free running text (one document per awardee) in an Excel workbook. Subsequently, the Excel workbook was imported into QDA Miner 5.0 (Provalis Research, Montreal, QC, Canada) for qualitative data analysis. The coding process started with an initial open-minded reading to learn the data. During the second reading, each narrative was scrutinised for emerging mutually exclusive categories or themes. A third read through was further performed after coding to fine-tune the results.

**TABLE 1:** Baseline data for World Association for the Advancement of Veterinary Parasitology African Foundation scholarship recipients who submitted 37 narratives to the World Association for the Advancement of Veterinary Parasitology Quarterly Online Newsletters for the years 2007–2017.

Variable	Category	No. of recipients	Percentage of recipients
Newsletter	Vol. 9 No. 4, November 2007	2	5.4
	Vol. 10 No. 1, April 2008	2	5.4
	Vol. 10 No. 2, October 2008	4	10.8
	Vol. 11 No. 2, December 2009	3	8.1
	Vol. 12 No. 1, April 2010	3	8.1
	Vol. 13 No. 2, October/November 2011	1	2.7
	Vol. 14 No. 1, April 2012	1	2.7
	Vol. 14 No. 2, November 2012	1	2.7
	Vol. 15 No. 2, October 2013	3	8.1
	Vol. 16 No. 1, April 2014	3	8.1
	Vol. 16 No. 2, October 14	1	2.7
	Vol. 16 No. 2, October 2014	1	2.7
	Vol. 17 No. 2, November 2015	2	5.4
	Vol. 18 No. 1, July 2016	3	8.1
	Vol. 19 No. 1, July 2017	1	2.7
	Vol. 19 No. 1, July 2017	4	10.8
	Vol. 19 No. 2, November 2017	2	5.4
Conference	2007: 21st International WAAVP Congress (Ghent, Belgium)	8	21.6
	2009: 22nd International WAAVP Congress (Calgary, Canada)	6	16.2
	2011: 23rd International WAAVP Congress (Buenos Aires, Argentina)	3	8.1
	2013: 24th International WAAVP Congress (Perth, Australia)	8	21.6
	2015: 25th International WAAVP Congress (Liverpool, United Kingdom)	5	13.5
	2017: 26th International WAAVP Congress (Kuala Lumpur, Malaysia)	7	18.9
Gender	Female	21	56.8
	Male	16	43.2
Country of origin	Egypt	1	2.7
	Ethiopia	2	5.4
	Kenya	3	8.1
	Nigeria	7	18.9
	South Africa	15	40.5
	Sudan	4	10.8
	Tanzania	1	2.7
	Tunisia	1	2.7
	Uganda	1	2.7
	Zambia	2	5.4

WAAVP, World Association for the Advancement of Veterinary Parasitology.

Two on-line databases (NCBI PubMed.gov and Web of Science) were accessed to determine the published research output of all 80 WAAVP AF awardees from 1999 to 2017. Research outputs included publications in high-impact peer-reviewed scientific journals in which these 80 awardees had published (Table 3).

### Ethical considerations

Ethical clearance was not required for the study.

## Results

Data for recipients originated from 11 WAAVP African Foundation Biennial Report and Financial Statements (1997–1999, 2001, 2003, 2005, 2007, 2009, 2011, 2013, 2015, 2017, 2019) which have been submitted to the WAAVP Executive Committee (https://www.waavp.org). Qualitative data analyses were extracted from the recipients’ narratives of WAAVP conferences and included nine categories/themes, frequency, and percentages (Table 2). The WAAVP AF scholarship announcement is sent biennially to over 100 global institutions to promote this opportunity.

**TABLE 2:** Nine emerging categories/themes were identified from 37 narratives in which recipients expressed their views pertaining to the World Association for the Advancement of Veterinary Parasitology African Foundation scholarships and attendance at the respective conferences 2007–2017 (*n* = 37).

Theme	Frequency	Percent
1. Expressed gratitude to WAAVP AF and WAAVP for the opportunity	31	83.8
2. Gained positive learning experiences	28	75.7
3. Were exposed to current research	22	59.5
4. Grew networking opportunities	19	51.4
5. Enjoyed interactions with other scientists	16	43.2
6. Increased learning of new skills	9	24.3
7. Received valuable feedback about their research presentations	8	21.6
8. Shared curriculum vitae and biographies with other scientists	4	10.8
9. Offered suggestions to the WAAVP conference organisers	2	5.4

WAAVP AF, World Association for the Advancement of Veterinary Parasitology African Foundation.

### Nine categories/theme details with examples of World Association for the Advancement of Veterinary Parasitology African Foundation award recipient excerpts

#### Theme 1: Expressed gratitude to World Association for the Advancement of Veterinary Parasitology African Foundation and World Association for the Advancement of Veterinary Parasitology for the opportunity

Almost all recipients of the WAAVP AF scholarships (83.8%) expressed gratitude for the invaluable support. Below are some excerpts in their own words:

‘THUMBS UP TO WAAVP; WAAVP FOR LIFE!’

‘I was an awardee of 2013 WAAVP AF Scholarship after a very tough completion among other African students. I am thankful that this scholarship catered for all my welfare including a round trip, accommodation and registration which I couldn’t manage to raise by myself.’ (Participant 25, Veterinary Parasitologist)

‘Therefore, I would like to take this opportunity to express my gratitude to the WAAVP African Committee for allowing me to benefit from such a prestigious scientific opportunity and the award of the travel scholarship.’ (Participant 12, Veterinary Parasitologist)

#### Theme 2: Gained positive learning experiences

More than three-quarters of the participants indicated that they were delighted to travel and that the trip was worthwhile the effort. Below are some excerpts in their own words:

‘From the opening ceremony to the Gala dinner, the social functions were splendid and enjoyable. What a great idea to have a fun run. As an enthusiastic runner myself, I used this as an opportunity to run wearing my Pretoria Marathon Club colours on another continent’. (Participant 10, Veterinary Parasitologist)

‘The social program, graced with spectacular and divine dishes, offered a great opportunity for networking. Other than the good food, which I enjoyed I must say, the perfect choice of the social event venues created a pleasant opportunity to visit the city’s greatest attractions’. (Participant 4, Veterinary Parasitologist)

‘All in all, it was a fantastic week of science and socializing, and the memories will stay with me for many years to come’. (Participant 18, Veterinary Parasitologist)

#### Theme 3: Were exposed to current research

Twenty-two participants indicated that they appreciated an exposure to current and contemporary research. Below are some excerpts from their narratives:

‘The WAAVP conference experience enhanced my knowledge on the important aspects of parasitology and neglected and zoonotic diseases of economic importance globally. It raised an alarming concern of how environmental differences influence parasites and the diseases they transmit, and the possibilities of future changes because of global warming.’ (Participant 37, Veterinary Parasitologist)

‘From the amazing keynote speakers and parallel presentations, I was consistently presented with great opportunities to attend to a diversity of fantastic science and well-organised presentations from all over the world. This has broadened my horizons and given me research ideas.’ (Participant 26, Veterinary Parasitologist)

‘The scientific program was rich and varied, reflecting the current priorities in veterinary parasitology with several sessions devoted to anthelmintic resistance, development of new anthelmintic drugs, livestock management strategies in drug resistance and emerging parasitic diseases, and the epidemiology of parasites in different environments, food-borne parasites, zoonoses, vaccine development and diagnostic techniques.’ (Participant 11, Veterinary Parasitologist)

#### Theme 4: Grew networking opportunities

About 50% of the recipients saw opportunities to network. Excerpts from some narratives are presented below:

‘It also allowed me to extend and establish a good network with people working in different part of the world. I created new contacts that were in line with my research but many more with those who were interested in what I was doing. Some were very pleased and would very much love to work with me in the future.’ (Participant 28, Veterinary Parasitologist)

‘We met a reputable number of scientists whom are representatives of parasitology laboratories and experts from universities. Working together, we can help our country in many ways through the provision of diagnostics and conduction of collaborative research studies through our available funded programmes, or by formulation of new proposals and by trying to seek national and international grants.’ (Participant 14, Veterinary Parasitologist)

‘An international conference such as the WAAVP not only exposes you to new people and research but also inspires you to keep up with your challenges and continue to make relevant contributions to science. It was exciting to make new friends and have interactions with a network of professional colleagues.’ (Participant 30, Veterinary Parasitologist)

#### Theme 5: Enjoyed interactions with other scientists

Sixteen of the recipients appreciated the opportunity to interact with other scientists. Some of the excerpts are presented below:

‘I applied and was accepted to attend the early career researcher breakfast which was held on the Monday morning. This breakfast certainly was a highlight showing that there are endless opportunities around the world and that one just needs to apply oneself. The breakfast also gave me the opportunity to meet new young researchers and build our own collaborations that one day in the future may be beneficial.’ (Participant 22, Veterinary Parasitologist)

‘Perhaps one of the most important aspects of the WAAVP meetings is the fact that they bring together, in our case, scientists from all over the world. We were able to meet up with colleagues, coworkers and friends from Belgium, Denmark, France, Italy, Malaysia, Mexico, Netherlands, Scotland, Sweden, the USA, the West Indies and many more, in a relaxed atmosphere of excellent Belgian food, beef and chocolate. Funnily enough, for those of us from Africa, it seemed to be an opportunity to speak to each other!!’ (Participant 3, Veterinary Parasitologist)

‘We met a reputable number of scientists who are representatives of parasitology laboratories and experts from universities. Working together, we can help our country in many ways through the provision of diagnostics and conduction of collaborative research studies through our available funded programmes, or by formulation of new proposals and by trying to seek national and international grants.’ (Participant 14, Veterinary Parasitologist)

#### Theme 6: Increased learning of new skills

Nine of the recipients stated that they learned new skills. Some of the excerpts are presented below:

‘I found the scientific programme was very rich with knowledgeable new information regarding the parasites in a changing landscape, including topics such as: epidemiology diagnostic and molecular diagnostic procedures, drugs and drug resistance, zoonosis. food-borne parasites, strategic control. In our fight against parasites in Sudan we will benefit from this information and will start to re-correct the steps either in the field or in the research.’ (Participant 14, Veterinary Parasitologist)

‘For my research line, anthelmintic resistance, I attended presentations with new ideas as well as techniques and findings. Professionally, the obtained knowledge of the direct discussions, presentations and posters will help me in the running project and in the design of future work.’ (Participant 34, Veterinary Parasitologist)

‘As a postdoctoral fellow in the field of veterinary parasitology, attending this conference offered me the chance to meet with famous scientists of the field and discuss my research related problems and solutions. It was an opportunity for me to gather information on new technologies and instruments related to my research area.’ (Participant 28, Veterinary Parasitologist)

#### Theme 7: Received valuable feedback about their research presentations

Eight of the recipients appreciated feedback about their research and/or presentation. Excerpts are included:

‘My participation in the conference enabled me communicate my research findings to a wider forum, and above all formed collaborative links in my area of research with scientists from across continents. It also helped me in my approach to research, data presentation and dissemination and above all lecturing.’ (Participant 23, Veterinary Parasitologist)

‘I was encouraged by the presence of veterans in veterinary parasitology who showed interest in young scientists’ research and put things into perspective with their questions and comments in all the different sessions of the conference. Some of their comments, we have brought back to our institutions to improve our work so that it is more relevant and has high impact.’ (Participant 4, Veterinary Parasitologist)

‘Attending WAAVP 2007 provided a great opportunity for me to present and received feedback on the findings of my Ph.D. research at an international conference.’ (Participant 6, Veterinary Parasitologist)

#### Theme 8: Shared curriculum vitae and biographies with other scientists

Four of the participants presented their curriculum vitae and biography statements.

#### Theme 9: Offered suggestions to the World Association for the Advancement of Veterinary Parasitology Conference Organizers

Two of the participants made suggestions to the conference organisers.

### Published research output by awardees

The results of the two database searches (NCBI PubMed.gov and Web of Science) indicated that the 80 awardees had published 808 articles in journals indexed by these databases. Of these, 146 articles were published in 23 African journals (Table 3).

**TABLE 3:** Published research output (*n* = 146) of 80 World Association for the Advancement of Veterinary Parasitology African Foundation travel scholarship recipients in 23 African journals.

Journal	No. of articles	Impact factor
African Crop Science Journal	4	0.29
African Health Sciences	1	0.842
African Journal of Agricultural Research	1	0.263
African Journal of Aquatic Science	1	0.750
African Journal of Biotechnology	1	0.44
African Journal of Range and Forest Science	1	0.961
African Journal of Traditional, Complementary and Alternative Medicine	2	0.553
African Journal of Wildlife Research	3	0.731
African Zoology	7	0.761
Bulletin of Animal Health and Production in Africa	12	0
Ethiopian Veterinary Journal	1	0
Journal of the South African Veterinary Association	28	0.69
Kenya Veterinarian	3	0
Nigerian Journal of Natural Products and Medicine	2	0
Nigerian Veterinary Journal	4	0
Onderstepoort Journal of Veterinary Research	33	0.94
Ostrich, Journal of African Ornithology	1	0.662
Pan-African Medical Journal	1	0
Sokoto Journal of Veterinary Science	1	0
South African Journal of Botany	28	1.504
South African Journal of Science	1	1.191
Tanzanian Journal of Health Research	1	0
Tanzanian Veterinary Journal	9	0.29

**Total**	**146**	**-**

## Discussion

Between 1999 and 2019, 80 WAAVP African Foundation awardees from 18 African countries were competitively selected to receive travel scholarships to present their research findings at WAAVP Biennial Conferences. The selection criteria included being registered for a postgraduate degree, submission of abstracts of scientific results which were accepted by the WAAVP AF Foundation Management Committee and WAAVP Conference Scientific Committee because of their high quality and a clear indication of how the balance of the funding required to attend would be met by the awardee.

The analyses of the impact that the WAAVP conferences made on the awardees included nine themes. The top three were as follows: 83.8% expressed gratitude to WAAVP and WAAVP AF for the opportunity to attend the conference, 75.7% gained positive learning experiences and 59.5% were exposed to current research.

In addition, the awardees were encouraged to publish their research findings in high-impact peer-reviewed scientific journals in Africa as well as abroad. The results of the database searches indicated that the 80 awardees had published at least 808 articles in more than 100 journals. Of these, 146 articles (18.1%) were published in African journals. Fifteen of these journals have an impact factor. The majority of the latter (*n* = 89; 61.0%) were published in three journals: *Onderstepoort Journal of Veterinary Research* (*n* = 33), *Journal of the South African Veterinary Association* (*n* = 28) and *South African Journal of Botany* (*n* = 28). This clearly indicates that the two South African veterinary journals play a major role as publishing platforms for research results from all over sub-Saharan Africa. The 28 articles in the *South African Journal of Botany* all emanated from the Phytomedicine Programme of the University of Pretoria, South Africa. Although impossible to quantify, there can be no doubt that awarding NG African veterinary parasitologists the opportunity of attending a major international conference and presenting their research data contributed significantly in stimulating their research output.

The WAAVP AF promises to continue for the foreseeable future. It attributes its 22 years of success to being financially well invested in South Africa, a dedicated WAAVP AF Committee, a fully supportive WAAVP Executive Committee, and awardees committed to identify innovative and novel solutions to address parasites that plague animal and human health.

This Foundation is unique and critical now and for the future of African veterinary parasitologists. We are encouraged that to date over 200 young scientists researching parasites of importance in Africa have applied from 25 countries, and many are now promoting their NG graduate students and postdoctoral students evidenced by the excellent output of research articles in peer-reviewed scientific journals.
